# Food Environments and Diet Quality Among Vendors and Consumers in Five Traditional Urban Markets in Kenya

**DOI:** 10.3390/nu17010116

**Published:** 2024-12-30

**Authors:** Kathrin M. Demmler, Sophie van der Steen, Ann Trevenen-Jones, Emilie de Kanter

**Affiliations:** 1 Global Alliance for Improved Nutrition, 10961 Berlin, Germany; 2 Independent Researcher, 2625 WL Delft, The Netherlands; 3 Global Alliance for Improved Nutrition, 3511 MH Utrecht, The Netherlands; 4 Julius Center for Health Sciences and Primary Care, University Medical Center Utrecht, 3584 CG Utrecht, The Netherlands

**Keywords:** traditional food markets, diet quality, urban food systems, nutrition policy, low- and middle-income countries, Kenya

## Abstract

Background/Objectives: Traditional food markets are essential in urban food environments in Kenya and other low- and middle-income countries (LMICs). They provide affordable fresh food, particularly for low-income urban communities, and are vital places of livelihoods and local economic activities. Despite their importance, associations between market-related factors and diet quality for vendors and consumers are underexplored. This study explores these relationships to inform policies aimed at improving diets and nutrition in LMICs. Methods: Survey data were collected from 1042 vendors and 876 consumers in five urban markets in Kenya. The survey assessed market-related factors, consumer purchasing behavior, socioeconomic factors, and dietary outcomes using the Kenya Diet Quality Questionnaire. Linear regression models were employed to evaluate associations between the availability of foods, consumers’ purchase of foods, proximity to the market, reported sickness from food, and diet quality indicators, including the Dietary Diversity Score (DDS), Global Dietary Recommendations (GDRs), NCD-Protect, and NCD-Risk scores. Results: Vendors’ own dietary outcomes were characterized by lower DDS, NCD-Protect, and NCD-Risk scores but higher GDR scores compared to consumers. Significant associations were identified between the purchase of specific food groups (e.g., vegetables, legumes, and nuts) and improved diet quality for consumers. Longer travel times were linked to lower diet quality for both vendors and consumers. Socioeconomic factors, such as gender, age, and education, significantly influenced diet quality. Conclusions: Traditional markets play a pivotal role in urban food environments. Policies that enhance market access and support vendors and consumers, particularly women, young adults, and low-income groups, are essential to improving diets and nutrition outcomes in LMICs.

## 1. Introduction

The burden of malnutrition poses significant threats to the health, well-being, and livelihoods of populations worldwide, costing trillions to health systems and economies [1,2]. Diets lacking essential micronutrients or excessively high in sugar, fat, or salt contribute to various forms of malnutrition, including undernutrition, micronutrient deficiencies, overweight, and related non-communicable diseases (NCDs). Despite widespread knowledge of these risks, significant barriers prevent many populations from accessing healthy diets, particularly in low- and middle-income countries (LMICs), where poverty remains pervasive [3].

The food environment, defined by factors such as availability, affordability, convenience, promotion, quality, and sustainability, plays a critical role in shaping food access and dietary behaviors [4,5,6]. This framework guides understanding and the development of strategic food environment interventions within markets, where vendors and consumers routinely connect around food. Besides other market-related factors, this paper focuses on physical aspects of the food environment, particularly proximity to traditional markets and food access.

In many LMICs, particularly urban and peri-urban areas, traditional food markets are a vital part of food environments. They are dynamic centers of trade, providing space and opportunities for small farmers, local businesses, entrepreneurship, and cultural food relationships, as well as stimulating growth and fostering resilience [7,8,9]. Rapid urbanization is driving the nutrition transition and socio-economic development in many LMICs [5,10,11]. While the expansion of formal retail outlets and supermarkets has become synonymous with urban development [12], traditional markets continue to be a key source of food and place of livelihoods, especially for low-income communities. Traditional markets mostly offer unprocessed fresh foods, such as fruits, vegetables, cereals, and legumes, ensuring food and nutrition security for urban populations, particularly those with limited financial buying power [7,13,14,15,16]. While important places of food, traditional markets face major challenges, such as inadequate infrastructure and basic services provision, informal regulatory mechanisms and variable enforcement thereof, as well as a lack of inclusion in food systems and governance decision-making [7,17,18,19].

Research on LMIC food environments is growing, but traditional food markets remain understudied [20,21]. Studies have highlighted the importance of food environment factors—such as accessibility, convenience, affordability, and food safety—in influencing consumers’ food choices and dietary behavior [22,23,24,25,26]. However, there is a significant lack of studies that assess quantitative relationships between market-related factors in traditional food markets and dietary outcomes in LMICs. Furthermore, rigorous, high-quality research studies in this context remain scarce. Notably, existing studies predominantly focus on consumers, neglecting the perspectives and diets of vendors and other key actors in the food environment.

This study addresses these gaps by examining market-related factors of traditional food markets in Kenya, and their associations with the dietary quality of market vendors and consumers. Focusing on both groups is essential, as vendors shape the market environment through the types and quality of the foods they offer, while consumers’ dietary quality reflects their access to and availability of these options. By analyzing both, the study provides a comprehensive understanding of market-related factors and the dynamic interactions between food supply and demand. In detail, the study’s objectives are to examine the associations between dietary quality (for both vendors and consumers) and the following:

- The types of food groups sold by vendors in traditional markets;
- The types of food groups purchased by consumers in traditional markets;
- The proximity to the market, as measured by travel time, for both vendors and consumers;
- The frequency of market visits by consumers;
- Reported experiences of food-related sickness or diarrhea among vendors and consumers.

Kenya presents an interesting context for studying urban communities and traditional food markets, due to its blend of traditional and rapidly modernizing food systems [27]. It is an LMIC [28] with a rapidly increasing urban population and a rise in overweight, obesity, and NCDs among adults, alongside persistent undernutrition in children [29]. On average, about two-thirds of food in Kenya is sourced informally, including through traditional food markets, kiosks, and street vendors [30].

## 2. Materials and Methods

### 2.1. Study Design

**Figure 1:**
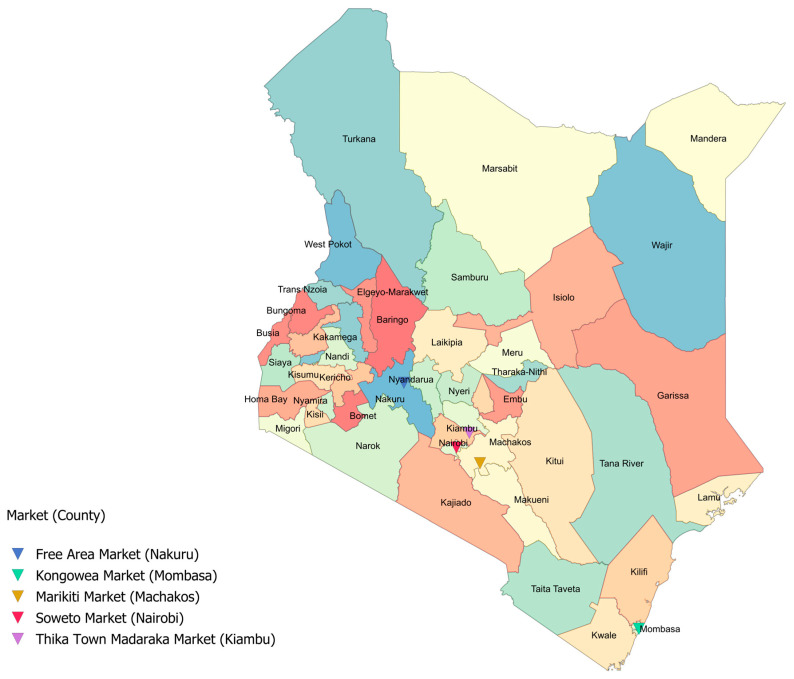
Map showing the location of the five traditional food markets and counties in Kenya. The map was created using ArcGIS^®^ software version 10.8 by Esri.

This cross-sectional study was conducted in 2022–2023 across five traditional food markets in urban Kenya. The study areas span from the western to the eastern parts in central-southern Kenya, including the markets Marikiti (Machakos County), Free Area (Nakuru County), Madaraka (Kiambu County), Kongowea (Mombasa County), and Soweto (Nairobi County) (Figure 1). While the markets were different with respect to their local context, such as the urban resident population surrounding the market, ecosystems, and local agricultural production and fishing, all of them were purposively selected due to their similarities and existing relationships with the Global Alliance for Improved Nutrition (GAIN). Specifically, two markets (Marikiti and Madaraka) were part of GAIN’s Keeping Food Markets Working (KFMW) program, which aimed to enhance more inclusive food systems governance and market resilience during and beyond COVID-19. Data collection for this study was conducted in two phases. The Marikiti market (Machakos town, Machakos County) served as a pilot location due to initial funding constraints. This allowed the refinement of study tools and procedures, ensuring their effectiveness before scaling up the study. Following the pilot, data collection was expanded to the remaining markets (Free Area, Madaraka, Kongowea, and Soweto), after securing additional funding.

All markets were of comparable sizes and infrastructure conditions, i.e., they were fairly large in expanse with a mix of covered and open-air market space. Additionally, the vendor density was comparable between the markets. All markets had some form of formal or informal market management and governance, typically linked to a market committee or association, as well as mandated governance relationships between county governments and markets and other levels of government (Table 1). The available food differed according to local context; however, all markets sold a wide selection of fresh fruits and vegetables, grains and pulses, as well as animal-source foods including dairy, eggs, meat, and fish.

### 2.2. Sampling

A random sampling approach was used to select both vendors and consumers within the markets. Sample size calculations were based on population estimates provided by market committees, which included both vendors operating within the market boundaries and consumers frequenting the markets on regular and market days. During the first phase of the study in the Marikiti market, a confidence level of 75% was applied for consumers to account for resource limitations at the time. Following the successful pilot phase, confidence levels were standardized to 85% for vendors and 80% for consumers to ensure consistency and comparability across the remaining four markets (Free Area, Madaraka, Kongowea, and Soweto). The target sample size was 964 vendors and 807 consumers, with an additional 8–10% oversampled to account for potential attrition (Supplementary Material, Table S1). In total, 1042 vendors and 876 consumers were surveyed.

Sampling within each market followed a systematic approach: the “Right Hand Rule” was used to select participants from randomly chosen entry points, targeting every fourth individual along the market route. While some clustering of specific food types was observed in certain areas (e.g., green leafy vegetable stands), most vendors were not spatially clustered by specific food types. Vendors selling only non-food items, and vendors and consumers below the age of 18 years were excluded. Data were collected at varying times of the day and week to ensure representativeness.

### 2.3. Data Collection

Data were collected in August 2022 (Marikiti Market (Machakos County)) and between April and May 2023 (in the four other markets) using structured surveys administered in Kiswahili and English. The surveys covered socio-economic and demographic information, food safety practices, and dietary behaviors. For vendors, questions related to business operations, seasonal availability of food, storage, and interest in nutrition training were included. For consumers, data on purchasing habits, frequency of market visits, and perceptions of market environments were collected. Dietary data for both vendors and consumers were captured using the Diet Quality Questionnaire (DQQ) adapted for Kenya [33]. The DQQ is a standardized, non-quantitative food frequency questionnaire that gathers information on the consumption of 29 sentinel food groups of the previous 24 h. Given its non-quantitative nature, the assessment is quicker, easier, and more cost-effective to administer, especially in large population surveys, compared to more resource intense tools like a 24 h dietary recall [34]. With the ability to derive meaningful dietary indicators from the assessment, the DQQ displays a valuable tool particularly for assessing diet quality in LMIC contexts [35,36].

#### 2.3.1. Dietary Data

The Dietary Diversity Score (DDS) reflects dietary diversity, based on the consumption of ten food groups (grains and tubers; pulses; nuts and seeds; dairy; meat, poultry and fish; eggs; dark green leafy vegetables; other vitamin A rich fruits and vegetables; other vegetables; and other fruits) of the previous 24 h, with scores ranging from 0 to 10 [37]. Higher scores indicate greater dietary diversity and have been linked to nutrient adequacy by several studies [38,39,40].

The Global Dietary Recommendations (GDRs) score measures adherence to global dietary recommendations as defined by the World Health Organization, which promote the consumption of protective food groups (e.g., whole grains, fruits, vegetables) and limit harmful dietary factors (e.g., processed meats, sugary beverages) [41]. The GDR score comprises two sub-components: NCD-Protect and NCD-Risk scores, which range from 0 to 9. A higher score suggests either a diet that protects against (NCD-Protect) or increases the risk of non-communicable diseases (NCD-Risk). The overall GDR score combines both sub-components by subtracting the NCD-Risk from the NCD-Protect and adding the value of 9. This provides a score ranging from 0 to 18, with higher scores reflecting better compliance with global dietary recommendations [42]. An overview of the diet quality indicators, their ranges, and descriptions can be found in the Supplementary Material, Table S2.

#### 2.3.2. Market-Related Factors and Consumer Purchasing Behavior

From a list of 29 sentinel food groups derived from the DQQ, the number of groups was reduced to 18 to simplify data collection and better reflect the local market context. This modification included practical considerations, such as the inclusion of bottled water, alcoholic drinks, and culinary ingredients, which were relevant to the market environment. For analysis purposes, these 18 food groups were further categorized into seven main groups: cereals; roots and tubers; legumes, seeds and nuts; vegetables; fruits; animal-source food (ASF); and miscellaneous, with the latter including culinary ingredients, sweets, and snacks (Supplementary Material, Table S3). The “miscellaneous” group, while heterogeneous, was retained to capture diverse foods relevant to the local context. This group includes items that may be associated with protective, risk-related, or “neutral” dietary behaviors, such as salt or other condiments. Vendors were asked to specify the types of foods typically sold in their businesses in the markets over the past 30 days, while consumers reported the types of foods they typically purchased in the same period.

Further independent variables included the average travel time to the market, reported in ranges of minutes. Participants reported their travel time based on their chosen mode of transport, which primarily included walking or public transport (e.g., matatus, shared minivans). For vendors, this variable reflects the time used to reach their workplace and hence the amount of time available for other tasks, e.g., related to food purchasing and preparation. For consumers, the travel time to the market, along with the frequency of market visits over the last 30 days, provides insights into the physical ease of reaching the markets and obtaining food.

Lastly, reported experiences of food-related sickness or diarrhea were assessed by asking both groups whether they knew someone who had become seriously ill from eating food in the past two years, providing insights into perceived food safety risks.

### 2.4. Statistical Methods

Statistical analyses were conducted using Stata version 15 (StataCorp, College Station, TX, USA). Associations between market-related factors, consumer purchasing behavior, and diet quality were analyzed separately for vendors and consumers using ordinary least square (OLS) regression models. The OLS model was chosen due to the nature of the diet quality scores, which were treated as continuous variables given their relatively large range (10, 11, and 19). This allowed us to focus on the overall variation in dietary diversity and quality, aligning with the study’s objectives. The normality of residuals was assessed using Skewness/Kurtosis and Shapiro–Wilk tests. While two of the four dependent variables exhibited significant deviations from normality, the sample size (n = 902 for vendors; n = 835 for consumers) ensured that OLS estimates remained unbiased and consistent [43]. Robust standard errors were applied to account for potential heteroskedasticity, and the models adjusted for individual and household characteristics such as gender, age, education level, household size, as well as vendors’ average sale values, receiving a business credit or loan, and consumers’ market spending, respectively. Standard errors were clustered at the county level to account for within-county correlations.

For the regression analyses, a limited sample size of 902 of the 1042 vendors and 835 of the 876 consumers was used, as data on vendors’ monthly sales values and consumers’ spending patterns were not available for the entire sample. These variables were deemed important for capturing key aspects of vendor and consumer behavior and significantly improved the model fit, as indicated by higher R^2^ values.

To address potential deviations from normality in the continuous dependent variables, an ordinary logit regression was also employed as a sensitivity analysis. This alternative specification confirmed the consistency of the OLS results, further reinforcing the robustness of the findings. The results of the ordinary logit regressions are presented in Supplementary Tables S4 (vendors) and S5 (consumers).

In addition to the regression analysis, differences between vendors and consumers were assessed using two-sample t-tests for continuous variables and z-tests for proportions in binary variables. These tests were employed throughout the study to provide descriptive comparisons that underline and complement the regression analysis results, with significance levels indicated at 10%, 5%, and 1%.

During the preparation of this manuscript, ChatGPT 4.0 was utilized to enhance the readability and clarity of the text. Following the use of this tool, the authors thoroughly reviewed and edited the content to ensure its accuracy and alignment with the intended scientific context.

## 3. Results

A total of 1042 vendors and 876 consumers were included in the descriptive analyses. For continuous variables, mean values and standard deviations (sd) are presented, while for binary variables, proportions with corresponding 95% confidence intervals (CIs) are provided. This descriptive summary of dietary quality and explanatory variables, disaggregated by vendors and consumers, is shown in Table 2.

Overall, the diet quality scores among respondents were high. The average DDS was 7.2, which was significantly lower for vendors (7.1) than for consumers (7.4). The most pronounced differences between vendors and consumers with a higher DDS (DDS ≥ 8) and vendors and consumers with a lower DDS (DDS ≤ 7) were observed in the consumption of eggs, meat, poultry, fish, pulses, nuts, seeds, and vitamin A-rich fruits and vegetables. Vendors had significantly lower NCD-Protect (5.1) and NCD-Risk scores (2.0), and a significantly higher mean GDR score (12.1) compared to consumers (11.3). Since the NCD-Risk score is a negative indicator, a lower mean value indicates a lower consumption of foods that increase the risk for NCDs, e.g., soft drinks, sweets, and highly processed foods.

Most consumers (58.9%) of this study relied on traditional food markets for all or most of their food purchases. Those consumers not exclusively going to the traditional markets (n = 360) mentioned also shopping at supermarkets (58.3%), roadside vendors (35.2%), kiosks (32.7%), or small/medium-sized shops (27.2%). Interestingly, consumers who exclusively shopped at traditional markets (n = 516) had significantly better diet quality, i.e., higher GDR scores by 0.3 points, compared to those who also used other food sources. This dietary improvement was primarily driven by a higher consumption of pulses, vitamin A-rich vegetables, citrus fruits, and unprocessed meat, alongside a lower consumption of baked grain-based sweets, fast food, and instant noodles. Conversely, those who supplemented their food purchases with supermarket shopping had a significantly higher consumption of whole grains but also a higher consumption of fast food, instant noodles, and baked grain-based sweets, and a lower consumption of pulses.

### 3.1. Market-Related Factors, Consumer Purchasing Behavior, and Diet Quality

Table 3 and Table 4 provide the results of the OLS estimates on the associations between foods sold and purchased at the market, time and frequency of market visits, reported experiences of sickness related to food, and vendors and consumers’ diet quality, respectively.

#### 3.1.1. Food Sold and Purchased in the Market

The food groups sold in the market by vendors had few significant associations with the vendors’ diet quality. Vendors selling ASF, including products like milk, dairy, poultry, fish, meat, and eggs, were associated with better diet quality, i.e., a significantly higher GDR score by 0.6 ‘points’ (Table 3). For this study, ‘points’ refers to the unit change in the dependent variable as estimated by the OLS regression coefficients. We did not find any significant differences in the food groups consumed by vendors selling ASF compared to other vendors. However, vendors selling vegetables had a significantly higher consumption of dark green leafy vegetables and vendors selling fruits had a significantly higher consumption of citrus fruits.

Consumers typically buying roots and tubers, legumes, seeds and nuts, and vegetables were significantly associated with having a better dietary diversity and quality, i.e., higher DDS, GDR, and NCD-Protect scores. In particular, consumers purchasing vegetables had the strongest relationship and a higher dietary diversity and better diet quality through the consumption of protective foods, i.e., higher DDS and NCD-Protect scores by 0.8 points each. On the other hand, consumers who typically purchased cereals were linked to lower diet quality through the increased consumption of foods that amplified the risk for NCDs, i.e., a higher NCD-Risk score by 0.3 points. Also, those purchasing fruits were associated with a significantly lower dietary diversity, i.e., a lower DDS by 0.2 points (Table 4).

#### 3.1.2. Time and Frequency of Market Visits

The travel time from home to the market presented significant associations with both vendors’ dietary diversity and consumers’ dietary quality, respectively; longer travel times were associated with an overall lower diet quality for both groups in these domains. Compared to those vendors spending less than 5 min to travel from home to the market, vendors taking between 5 and 10 min, 10 and 20 min, 30 and 60 min, and more than 60 min experienced notably lower dietary diversity, i.e., a decreased DDS between 0.8 and 1.0 points (Table 3).

For consumers, the relationship between travel time and diet quality appeared to be almost negatively linear, i.e., a higher travel time was related to lower diet quality through the consumption of food protective against NCDs (NCD Protect), but also led to a higher diet quality through the lower consumption of foods associated with NCDs. Consumers spending 5–10 min traveling to the market, compared to less than 5 min, showed the beginning of reductions in diet quality scores. These reductions were most pronounced for those traveling 60 min or more, with decreases of 1.1 points and 1.5 points, in GDR and NCD-Protect scores, respectively, alongside a decrease in dietary diversity (DDS) by 1.0 points (Table 4). Although travel time negatively affected consumers’ consumption of different food groups, i.e., the consumption of food groups protective against NCDs and those recommended for limited consumption, it did not affect the GDR score.

The regression results in Table 4 indicate that compared to consumers visiting the market several times per day, those visiting once per week had significantly higher dietary diversity (DDS) by 0.8 points. However, it was difficult to discern a clear trend between the frequency of market visits and consumers’ diet quality, with only the DDS showing significant associations. Those visiting once per week consumed significantly higher shares of cereals and tubers, meat, poultry and fish, dark green leafy vegetables, and other vitamin A-rich fruits and vegetables.

Surprisingly, the frequency of market visits did not significantly decrease with the longer travel times of the consumers; even among those spending more than 60 min to reach the market, half continued to do most of their shopping there. This observation was reinforced by a weak correlation (Pearson correlation = 0.29) between consumers’ travel time and market visiting frequency.

#### 3.1.3. Reported Experiences of Sickness Related to Food

Vendors that reported experiencing diarrhea and sickness related to food over the course of the last 2 years did not show significant associations with any diet quality outcome in the regression analysis. However, consumers that reported experiencing diarrhea and sickness related to food were associated with significantly better dietary diversity, i.e., a higher DDS by 0.5 points.

### 3.2. Socioeconomic and Demographic Factors and Diet Quality

Compared to their male counterparts, female vendors were associated with a significantly lower diet quality related to the higher consumption of foods that increase the risk for NCDs, i.e., they had a higher NCD-Risk score by 0.3 points. Also, female consumers exhibited a significantly lower diet quality, i.e., lower GDR scores by 0.5 points.

**Figure 2:**
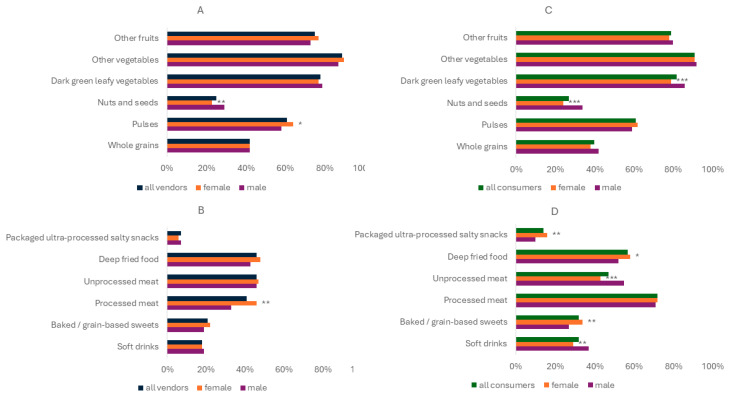
Share of selected food groups consumed by vendors (n = 1042) and consumers the prior 24 h, by gender. (**A**) Dietary factors protective of NCDs, vendors. (**B**) Dietary risk factors for NCDs, vendors. (**C**) Dietary factors protective of NCDs, consumers. (**D**) Dietary risk factors for NCDs, consumers. * Significant at 10% level; ** Significant at 5% level; *** Significant at 1% level.

Figure 2 depicts that female vendors consumed significantly more pulses and processed meat, while they consumed less nuts and seeds the prior 24 h. Additionally, female consumers consumed significantly more packaged ultra-processed and salty snacks, deep-fried food, and baked/grain-based sweets, while consuming less dark green leafy vegetables, nuts and seeds, unprocessed meat, and soft drinks.

Compared to the youngest vendor group aged 18–24 years, vendors in the age groups 51–65 years and 66–75 years were associated with a significantly higher overall diet quality and lower consumption of foods that increase the risk of NCDs, i.e., a higher GDR score by 1.2 points and 1.3 points, respectively, and a lower NCD-Risk score by 1.2 points and 1.6 points, respectively. However, vendors aged 51–65 years and 66–75 years were also associated with significantly lower dietary diversity, i.e., a lower DDS by 0.4 points and 0.9 points, respectively (Table 3). Compared to consumers aged 18–24 years, those consumers aged 25–30 years, 31–40 years, and 66–75 years were associated with better dietary diversity, i.e., a higher DDS by 0.6 points, 0.7 points, and 1.6 points, respectively. Those aged 31–40 years and older were associated with a better overall diet quality and lower consumption of foods that increase the risk for NCDs, i.e., higher GDR scores between 1.3 points and 3.8 points, respectively, and lower NCD-Risk scores between 0.6 points and 3.1 points, respectively, with the strongest effect size in the age group of 66–75-year-olds. Furthermore, those aged 66–75 years were associated with an increased consumption of foods protective against NCDs, i.e., an increased NCD-Protect score of 0.7 points (Table 4).

No significant associations were observed between vendors’ educational attainment and any of the diet quality outcomes. Consumers who had completed primary education, compared to those with no formal education, were associated with a better overall diet quality and a lower consumption of foods that increase the risk of NCDs, i.e., higher GDR and NCD-Protect scores by 1.0 points and 0.5 points, respectively.

Neither the vendors’ average monthly values of food sold in the market nor accessing credit or loan showed any significant associations with vendors’ diet quality. Consumers who spent between KES 100–<200 (3–<5 PPP) and KES 200–<300 (5–<7 PPP) at the market per visit had significantly lower dietary diversity (DDS) by 0.8 points and 0.5 points, respectively, compared to those spending less than KES 100 (3 PPP). Additionally, those in the KES 100–<200 (3–<5 PPP) spending bracket were significantly associated with a lower consumption of food protective against NCDs, i.e., a decreased NCD-Protect score by 1.0 points (Table 4).

Household size did not demonstrate significant associations with any of the diet quality indicators for either vendors or consumers.

## 4. Discussion

This study provides valuable insights as it is, to our knowledge, the first to quantify the association between market-related factors, consumer purchasing behavior, and diet quality outcomes for both vendors and consumers in urban areas of Kenya.

### 4.1. Diet Quality in Urban Kenya

In Kenya, as in many other rapidly urbanizing LMICs, there is a critical shift in dietary habits leading to an increase in overweight, obesity, and nutrition-related NCDs [45,46,47]. This makes it crucial to understand the factors influencing dietary outcomes from both public health and policy perspectives, aiming to improve the local food environment and enhance food systems.

Our findings revealed higher DDS and NCD-Protect scores among vendors and consumers compared to data from the Diet Quality Project for urban Kenyan populations in 2021, with differences of 1.5 points and 1.2 points, respectively [48]. The GDR scores and NCD-Risk scores were relatively comparable, showing differences of 0.7 points and 0.2 points, respectively. As both studies used the same tool, the Diet Quality Questionnaire (DQQ), to assess dietary quality, these comparisons are valid. The observed improvements could reflect a recovery in dietary quality post-COVID-19 pandemic, or they may be influenced by seasonality or differences in the study population. While our sample focused on vendors and consumers in traditional food markets, who may be comparatively better off than the general urban population, it is also possible that these differences indicate a positive trend in dietary quality over time.

### 4.2. Market-Related Factors, Consumer Purchasing Behaviour, and Diet Quality

Although some descriptive results find connections between the types of foods sold and consumed—for vendors selling vegetables or fruits—except for vendors selling ASF, there was no clear association between the types of food vendors sold and their diet quality, implying that their dietary outcomes did not directly correlate with the foods sold. In a study from urban Uganda, 71% of the foods consumed by street food vendors came from their own stalls [49]. For smallholder farmers in LMICs, on-farm production diversity generally linked strongly with dietary diversity [50]. However, depending on the context, access to markets was equally or sometimes even more crucial for dietary diversity [50,51,52]. These insights imply that for the included traditional food market vendors selling fresh produce—as opposed to street food vendors selling prepared meals or farmers consuming their own produce—dietary quality might be influenced more by convenience and socio-demographic factors than by the foods they sell.

An exception was noted for ASF. A panel data analysis of small-scale farmers in Uganda found better dietary diversity in terms of increased consumption of ASF, influenced by farmers’ own production rather than purchased foods [53]. However, it is difficult to transfer these findings to the market vendors. In this study, ASFs were included in the GDR score as a negative contributor, making up parts of the NCD-Risk score with processed and unprocessed meat. While the monthly sales values of vendors were a control factor in the regression analysis, 23.1% of vendors also had other income sources besides being a vendor at this market or other markets. Hence, it is likely that those selling ASFs might have indirectly benefitted from a higher diet quality, e.g., through overall higher incomes despite their monthly sales values from this market, leading to a diet richer in food protective against NCDs and lower in highly processed foods. Furthermore, the group of vendors selling ASFs had a rather small sample size (n = 42); hence, the interpretation of the results should be carried out cautiously.

While consumer purchases do not directly reflect a food environment factor, they provide insights into the accessibility of foods, combined with affordability factors and personal preferences. Furthermore, the agreement between purchases and dietary outcomes underscores the usefulness of the assessment methods applied for purchases and their translatability into dietary outcomes. Our findings reveal a strong connection between the regular purchase of vegetables, roots and tubers, legumes, seeds and nuts and improved diet quality among consumers. However, for purchasing cereals and fruits, the relationship with diet quality was negative. This outcome contrasts with the initial prediction that consumer purchases of these food groups would lead to neutral or positive associations with diet quality. Hence, the results indicate a moderate association and agreement between food group purchases and the individual diet quality of consumers through the given methods. Given that the assessed consumer purchases reflect household purchasing patterns over a 30-day period, the variability between purchases and diet quality might be explained. This is particularly relevant since the measures of diet quality in this study rely on a single assessment of the previous day and night. It is important to note that the reported food groups bought at the market reflect the respondent’s view and might be incomplete, as other household members may purchase additional food for the household or they might purchase additional food at other outlets. Furthermore, purchasing behavior is strongly correlated with seasonality and affordability, factors that this study could not control for and that likely influence the results. Overall, it is not expected that food purchases and dietary intake would demonstrate near-perfect agreement, even in theory [54].

Physical distance from the market played a critical role in both vendors’ dietary diversity and consumers’ overall diet quality. Similarly to the findings of this study on consumers, a study from urban Tanzania identified associations between a greater density of vegetable vendors within 500 m of the household and an increased likelihood of purchasing vegetables and lower energy intake [24]. Others found that proximity to supermarkets and other food outlets increased fruit and vegetable consumption in urban Brazil but did not affect the consumption of sugar-sweetened beverages [55]. Further, perceived proximity to fresh produce markets encouraged the consumption of minimally processed foods among women in Santos [56].

The expectation that a higher frequency of market visits leads to higher dietary diversity, e.g., through more perishable foods, is not supported by our results. We find that those visiting once per week compared to those visiting several times per day reported a higher consumption of perishable foods, including meat and fruits and vegetables, as well as the consumption of more staple foods, including cereals and tubers. Hence, these associations are presumably driven by unobservable socio-economic factors and habits. For example, those visiting once per week might have a more structured shopping approach and meal planning.

Overall, the results on the time and frequency of market visits underscore the importance of traditional food markets for both vendors and consumers. It was common for respondents to travel up to 30 min or more to reach the markets where they worked or bought food. For vendors, this suggests that the business opportunities in these markets justify the travel. For consumers, it indicates that options to buy fresh food elsewhere in the cities might be limited or less preferred for various reasons. The weak correlation between travel time and market visit frequency further indicated that the markets’ value to consumers transcends the inconvenience of longer travel distances, underscoring their critical role in urban food systems in Kenya.

Consumers who reported experiencing sickness from food over the last two years were associated with significantly higher dietary diversity. These results are surprising as other studies found more diverse diets to physiologically enhance gut health and immunity, contributing to better health outcomes [57]. However, in this context and given the nature of the question, which refers to the last two years, the identified associations might also reflect a heightened awareness or an effort to adopt healthier dietary practices following illness. This suggests a behavioral explanation rather than a biological one.

### 4.3. Socioeconomic and Demographic Factors and Diet Quality

Female vendors were associated with a significantly higher consumption of foods with dietary risk factors for NCDs (NCD-Risk), particularly processed meat. And they had significantly lower overall diet quality (GDR score), probably driven through a lower consumption of protective foods including dark green leafy vegetables and nuts and seeds, and an increased consumption of ultra-processed foods, deep-fried foods, and baked/grain-based sweets. These results differ from the latest diet quality data for Kenya [48]. Based on their findings, including urban and rural data from 2021, women had a lower NCD-Risk score and a higher GDR score, and did not show any differences between their NCD-Protect scores. However, evidence also shows that the national prevalence of overweight and obesity is significantly higher among women. Estimates for Kenya indicate a 29 percentage point higher prevalence of overweight and obesity for women in 2019 [58]. These findings could be partially explained by the unique challenges women face in urban settings, which may vary across contexts. While women in many LMICs and developed countries often play a central role in planning and preparing meals, their involvement in food purchasing can differ [59]. For example, in some contexts like Pakistan, men frequently handle routine shopping while women focus on meal preparation and household food planning. In urban areas, additional factors such as time constraints, resource limitations, and cultural norms may further influence dietary choices, potentially leading to suboptimal diets. This dynamic may be exacerbated by the higher prevalence of food consumption away from home, which can disproportionately affect dietary outcomes. Evidence from a study on women and their food choices in informal settings in Kenya underlined that factors like food affordability and time were often seen as trade-offs for nutrition, leading to sub-optimal diets [25]. Hence, the results of this study might not be too far off from reality and contribute to understanding the underlying dietary differences, which may be influenced by gender-specific roles, responsibilities, and dietary habits.

Furthermore, age emerged as a significant factor for diet quality. For vendors, increasing age was associated with higher diet quality (GDR score) and a lower consumption of dietary risk factors for NCDs, as well as lower dietary diversity. The latter was most likely affected by larger decreases in foods with dietary risk factors, which, despite leading to higher diet quality, could impact the overall dietary diversity, negatively. This phenomenon indicates that while older vendors compared to those aged 18–24 years exhibit overall better dietary practices, their dietary diversity may not be as pronounced. For consumers, the association between age group, dietary diversity, and diet quality was positive. The strong associations are likely due to a combination of a reduced consumption of dietary risk factors, e.g., soft drinks, sweets, and highly processed foods, and an increased intake of foods protective against NCDs, e.g., fruits and vegetables, legumes, and whole grains. Overall, this aligns with the insights on vendors, which indicate that younger vendors and consumers had lower diet quality than older respondents. Studies from Brazil underline the factor of age showing better diet quality in older adults aged 60 years and over, compared to adolescents and younger adults [60].

This study further showed the importance of primary education in promoting compliance with dietary recommendations and the consumption of foods protective against NCDs within consumers. It further raises questions about the relative impact of primary education compared to secondary, tertiary, or vocational education on dietary behaviors.

### 4.4. Study Limitations

This study is constrained by several limitations. Firstly, the analyses are not designed in a manner that causal relationships can be determined. The use of cross-sectional data introduces inherent challenges, particularly the risk of overestimating the strength of observed associations. Additionally, the selection of markets for this study is constrained by the purposeful selection of traditional local food markets, with a focus on those linked to GAIN’s co-designed interventions. Even so, these markets are largely like other public food markets in these counties, and more broadly, the whole country. In terms of other markets, the area of market footprint, actual basic service delivery and infrastructure like WASH, and variations of food groups sold may differ. While our analyses consider these aspects and cluster standard errors by locality, this could influence the generalizability of the findings. While the sample sizes of vendors and consumers represent both groups within each traditional food market, the chosen confidence levels are a limitation. This was a necessary compromise to ensure the feasibility of the research within the available resources. Future studies might consider higher confidence levels to strengthen the robustness of the findings.

Income and expenditure are likely important factors in the association between market-related factors, consumer purchasing behavior, and diet quality. However, the indicators of vendor monthly sales values and consumer spending per visit do not capture the full range of income for vendors or expenditure for consumers. Although collinearity aspects have been carefully checked, the relationships between income and dietary outcomes, particularly for consumers, might be reflected in travel time results, as low-income consumers are more likely to live further away from the market. The inclusion of vendor monthly sales values and consumer spending patterns introduced another limitation: a substantial dropout of observations due to missing data on these aspects. However, since running the analyses with limited sample sizes and including the sales and spending insights still yielded a better model fit than dropping the variables, we kept them.

Due to the logistical challenges of collecting anthropometric measurements in a market setting, we were unable to include such data in this study. Future research should consider incorporating anthropometric data to establish more direct links between diet quality and nutritional outcomes, particularly in relation to non-communicable diseases.

These limitations underscore the importance of interpreting the study’s findings with caution. While this study investigates selected characteristics of traditional food markets, such as proximity and food access, it does not encompass the full breadth of food environment characteristics, such as socio-economic, political, or structural features. Future research should address these gaps by incorporating a more comprehensive assessment of food environment dimensions, including food supply (availability), affordability (accessibility), infrastructure market investment (accessibility and resilience), basic service delivery (accessibility), vendor marketing and credit offerings, consumer perceptions of markets, sustainability and socio-ecological insights, including culture, along with food systems governance. Additional data, longitudinal studies, and more diverse market sampling could further enrich our understanding of how food environments in traditional food markets influence dietary quality and nutrition outcomes in different contexts.

### 4.5. Policy Implications and Further Research Directions

This study highlights the critical role of traditional food markets and their market-related factors for providing access to diverse, safe, and nutritious foods, and significantly influencing the diet quality of both vendors and consumers in urban Kenya. Several policy implications emerge from these findings to enhance dietary quality in such contexts.

Firstly, there is a need for dietary information campaigns that effectively and inclusively target and engage both vendors and consumers. Educational programs can be designed to inform, skill, and empower vendors about food systems, nutrition, and food safety while also leveraging the routine vendor–consumer relationships in the markets to optimize the communication of such knowledge. Awareness and knowledge of healthy eating practices and the benefits of consuming diverse, nutritious, and safe foods can improve vendors’ own diet quality and that of their customers. Innovative education initiatives are needed; ones that are systemic, culturally and contextually adaptable yet also scalable, attentive to gender and the multiple vulnerabilities of urban low-income vendors and consumers, and engage in active learning rather than passive, top-down training. Sub-national governments, including county and local governments, can support these campaigns. This also involves their synergy with traditional food market-related resources and activities, across departments, such as those involved with trade, health, and agriculture. For example, common food systems and nutrition messaging across departments and leveraging community health workers and agricultural extension officers’ activities. Furthermore, such efforts should be combined with incentives for behavior change and the sale of diverse, nutritious, and safe food. These endeavors need to consider vendors’ existing practices and knowledge, and the importance of medium and long-term capacity building rather than one-off and/or short-term successes [61,62,63].

Policies should also address dietary risks, particularly among women and younger adults, by promoting healthier eating habits and increasing awareness of the benefits of consuming protective foods. These efforts require the active involvement of key stakeholders, including market vendors and local communities, through public consultations and stakeholder workshops to ensure responsive policy development and uptake in practice.

Urban planning can prioritize the spatial development and food distribution networks of local food outlets in underserved neighborhoods while also supporting traditional food markets as essential sources of diverse, safe, and nutritious foods. Policies and strategies should focus on enhancing market infrastructure, such as cold storage and waste management. These efforts could include de-risking financial investment models to fund such infrastructure, e.g., by reinvesting market fees, and empowering market committee leaders with the agency and governance tools needed to support the effective use and maintenance of these improvements [7,64,65,66].

Additionally, policies need to consider the broader role of various food outlets, including street food vendors, kiosks, grocery stores, and supermarkets, in shaping urban food environments and promoting nutritious diets. While increasing access to healthy ready-to-eat foods and regulating the availability and marketing of energy-dense, highly processed foods—regardless of their sources—are crucial, future studies could also compare findings with local food environments in developed economies. Despite differing socio-economic and cultural contexts, many shared characteristics transcend these differences, offering valuable insights for shaping urban food systems globally. Measures such as front-of-pack nutrition labeling, modeled on Mexico’s nutrition labeling law, could be adapted to the Kenyan context [67]. Furthermore, policies to improve food affordability, such as commodity-specific vouchers and the fortification of staple foods, could also contribute to better nutrition [68].

Finally, policies must be accompanied by regular monitoring of market-related factors, food safety standards, and dietary quality indicators. Tools like the DQQ can provide rapid and standardized assessment, while research focusing on underexplored aspects of the food environment, such as desirability and convenience, along with robust analyses using panel data, can help refine policies and strategies to enhance dietary outcomes across diverse population segments.

By leveraging traditional markets, promoting greater local agency, and involving vendors and consumers as active stakeholders in food systems’ transformation, these efforts can contribute significantly to healthier diets in urban Kenya and similar contexts in other LMICs.

## 5. Conclusions

This study underscores the pivotal role of traditional food markets in urban Kenya as critical elements of the food environment. By examining how market-related factors and consumer purchasing behavior shape both vendors and consumers’ diet quality in LMICs, the findings highlight the need for targeted policies and interventions to enhance the role of traditional markets in promoting healthier diets and improving nutritional outcomes.

## Figures and Tables

**Table 1:** Market characteristics.

	Markets				
Characteristic	Marikiti	Free Area	Madaraka	Kongowea	Soweto
County	Machakos	Nakuru	Kiambu	Mombasa	Nairobi
Urban center	Machakos	Nakuru	Thika	Mombasa	Nairobi city
Urban center population ^1^	64,000	570,000	250,000	1,200,000	4,400,000
Food poverty rate, county ^2^	31.8%	26.5%	18.3%	25.9%	15.8%
Market characteristics ^3^					
Number of vendors p. day	4000	450	1400–2000	4000	600
Number of consumers p. day	5000–10,000	800–1000	2000–3000	2500	5000
Share of those taking <20 min to reach the market (vendors and consumers) ^4^	58.0%	73.1%	71.0%	65.5%	87.4%
Market offers mainly fresh/raw foods	yes	yes	yes	yes	yes
Major food groups sold	Fruits, vegetables, cereals	Vegetables, fruits, roots and tubers	Vegetables, fruits, roots and tubers	Vegetables, fruits, roots and tubers	Vegetables, fruits, roots and tubers
Market ownership	Government-led	Government-led	Government-led	Government-led	Government and community-led
Market has a functional market committee	yes	yes	yes	yes	yes
Market operates daily	yes	yes	yes	yes	yes
Market location is fixed, with no plans to remove or significantly alter borders	yes	yes	yes	yes	yes
GAIN program links	KFMW	None	KFMW	None	None

^1^ Population size of the urban center as defined by the 2019 Kenya Population and Housing Census [31]. ^2^ The share of individuals in a county whose food consumption per adult equivalent was less than the food poverty line of KES 2668 per month in rural areas and KES 3520 per month in urban areas [32]. ^3^ Market characteristics were assessed through observations and insights shared by local market committees. ^4^ Insights are based on the vendor and consumer surveys. GAIN: Global Alliance for Improved Nutrition; KFMW: Keeping Food Markets Working program.

**Table 2:** Descriptive statistics by vendors and consumers.

			Vendor		Consumer	
Variable			Mean ± sd	freq. [95% CI]	Mean ± sd	Freq. [95% CI]
Diet quality (continuous)						
	Dietary Diversity Score (0–10)		7.05 *** ± 1.80		7.35 ± 1.66	
	Global Dietary Recommendations score (0–18)		12.10 *** ± 2.04		11.32 ± 2.31	
	NCD-Protect score (0–9)		5.05 *** ± 1.87		5.27 ± 1.75	
	NCD-Risk score (0–9)		1.95 *** ± 1.81		2.96 ± 2.27	
Female (1/0)				0.61 *** [0.58–0.64]		0.67 [0.64–0.7]
Age groups (1/0)						
	18–24 years			0.06 *** [0.05–0.08]		0.14 [0.12–0.16]
	25–30 years			0.19 *** [0.16–0.21]		0.32 [0.28–0.35]
	31–40 years			0.31 [0.28–0.34]		0.30 [0.27–0.33]
	41–50 years			0.26 *** [0.23–0.28]		0.17 [0.15–0.2]
	51–65 years			0.17 *** [0.15–0.20]		0.07 [0.05–0.09]
	66–75 years			0.02 *** [0.01–0.03]		0.00 [0.0–0.01]
Education, finalized (1/0)						
	No formal education			0.08 *** [0.06–0.09]		0.05 [0.03–0.06]
	Primary education			0.29 *** [0.27–0.32]		0.13 [0.11–0.16]
	Secondary education			0.48 [0.45–0.51]		0.45 [0.42–0.48]
	University education			0.06 *** [0.05–0.08]		0.21 [0.18–0.24]
	Technical/vocational education			0.09 *** [0.07–0.11]		0.16 [0.14–0.19]
Household size (continuous)			4.30 *** ± 1.90		3.99 ± 1.85	
Travel time to reach the market from home (1/0)						
	<5 min			0.04 [0.03–0.05]		0.03 [0.02–0.05]
	5–<10 min			0.30 ** [0.28–0.33]		0.26 [0.23–0.29]
	10–<20 min			0.37 ** [0.34–0.40]		0.41 [0.38–0.45]
	20–<30 min			0.19 [0.17–0.22]		0.21 [0.19–0.24]
	30–<60 min			0.07 [0.05–0.08]		0.06 [0.04–0.08]
	≥60 min			0.03 * [0.02–0.04]		0.02 [0.01–0.03]
Food groups sold/purchased at market, last 30 days (1/0) ^a^						
	Cereals			0.09 [0.07–0.11]		0.35 [0.32–0.38]
	Roots and tubers			0.18 [0.16–0.2]		0.47 [0.44–0.51]
	Legumes, seeds and nuts			0.07 [0.06–0.09]		0.27 [0.24–0.30]
	Vegetables			0.66 [0.63–0.69]		0.91 [0.89–0.93]
	Fruits			0.41 [0.38–0.44]		0.7 [0.66–0.73]
	Animal-source food			0.04 [0.03–0.05]		0.19 [0.16–0.21]
	Miscellaneous (ingredients, sweets, snacks)			0.03 [0.02–0.04]		0.05 [0.03–0.06]
Average monthly sales values from food, last 12 months (1/0) ^b^						
	KES < 15,000	<350 PPP		0.2 [0.18–0.23]		
	KES 15,000–<45,000	350–<1040 PPP		0.40 [0.37–0.43]		
	KES 45,000–<75,000	1040–<1730 PPP		0.21 [0.18–0.23]		
	KES 75,000–<105,000	1730–<2420 PPP		0.08 [0.06–0.10]		
	KES 105,000–<135,000	2420–<3120 PPP		0.05 [0.03–0.06]		
	KES 135,000–<165,000	3120–<3810 PPP		0.03 [0.02–0.04]		
	KES ≥ 165,000	≥3810 PPP		0.04 [0.03–0.05]		
Credit/loan received (1/0)				0.32 [0.29–0.35]		
Frequency of market visits, last 30 days (1/0)						
	Several times per day					0.03 [0.02–0.05]
	Daily, once per day					0.19 [0.16–0.22]
	Every 2–3 days					0.27 [0.24–0.30]
	Once per week					0.32 [0.28–0.35]
	Every two weeks					0.11 [0.09–0.13]
	Once per month					0.06 [0.04–0.08]
	Less than once per month					0.03 [0.02–0.04]
Money spent on food at the market each visit, last 30 days (1/0) ^c^						
	KES <100	<3 PPP				0.04 [0.02–0.05]
	KES 100–<200	3–<5 PPP				0.06 [0.05–0.08]
	KES 200–<300	5–<7 PPP				0.11 [0.09–0.13]
	KES 300–<400	7–<9 PPP				0.09 [0.07–0.11]
	KES 400–<500	9–<12 PPP				0.14 [0.12–0.17]
	KES 500–<1000	12–<23 PPP				0.22 [0.19–0.25]
	KES ≥1000	≥23 PPP				0.35 [0.32–0.38]
Doing all/most food shopping at the market (1/0)						0.59 [0.56–0.62]
Alternative food outlets used for food purchases, last 30 days (1/0) ^d^						
	Other wet markets					0.10 [0.07–0.13]
	Roadside/street vendors					0.35 [0.30–0.40]
	Supermarket/hypermarket					0.58 [0.53–0.63]
	Kiosk					0.33 [0.28–0.38]
	Small/medium shop					0.27 [0.22–0.32]
	Farm					0.04 [0.02–0.06]
	Own production					0.04 [0.02–0.06]
Diarrhea/sickness from food, last 2 y (1/0)				0.08 ** [0.06–0.10]		0.05 [0.04–0.07]
N			1042		876	

N, number of observations; sd, standard deviation; freq., frequency; CI, Confidence Interval. * Significant at 10% level; ** Significant at 5% level; *** Significant at 1%. (1/0) indicates a binary variable or binary categories of a variable, e.g., for age group, education, etc. ^a^ For vendors: food groups sold at the market; for consumers: food groups purchased at the market. ^b^ Data only available for n = 902 vendors. PPP conversion factor for 2023 is KES 43.29 per international dollar [44]. PPP values have been rounded to the nearest 10 for clarity. ^c^ Data only available for n = 835 consumers. PPP conversion factor for 2023 is KES 43.29 per international dollar [44]. PPP values have been rounded to the nearest whole number for clarity. ^d^ This question was only asked to consumers, who did not state to do all/most of their food shopping at the market (n = 360).

**Table 3:** Regression results for the associations of market-related factors of traditional food markets on vendors’ DDS, GDR score, NCD-Protect, and NCD-Risk score.

Variable			DDS	GDR	NCD-Protect	NCD-Risk
Food groups sold in the market, last 30 days						
	Cereals		−0.38 (0.45)	0.17 (0.38)	−0.09 (0.44)	−0.26 (0.17)
	Roots and tubers		0.16 (0.16)	0.37 (0.23)	0.26 (0.17)	−0.10 (0.30)
	Legumes, seeds and nuts		−0.12 (0.25)	−0.06 (0.36)	−0.05 (0.29)	0.01 (0.14)
	Vegetables		0.09 (0.14)	0.16 (0.25)	0.16 (0.14)	0.00 (0.24)
	Fruits		−0.05 (0.11)	0.06 (0.25)	0.16 (0.15)	0.10 (0.11)
	Animal-source food		0.10 (0.21)	0.55 ** (0.17)	0.32 (0.20)	−0.23 (0.30)
	Miscellaneous		0.10 (0.61)	0.50 (0.87)	0.30 (0.49)	−0.20 (0.49)
Travel time to the market						
	less than 5 min (reference)		0.00 (.)	0.00 (.)	0.00 (.)	0.00 (.)
	5–<10 min		−0.90 ** (0.27)	−0.21 (0.51)	−0.47 (0.35)	−0.27 (0.25)
	10–<20 min		−0.91 ** (0.24)	−0.45 (0.47)	−0.54 (0.32)	−0.09 (0.22)
	20–<30 min		−0.45 (0.25)	−0.04 (0.39)	0.05 (0.32)	0.08 (0.26)
	30–<60 min		−0.97 * (0.37)	−0.45 (0.48)	−0.73 (0.41)	−0.27 (0.31)
	60 min/1 h and more		−0.78 * (0.29)	−0.46 (0.51)	−0.52 (0.37)	−0.06 (0.30)
Diarrhea/sickness from food, last 2 y			−0.49 (0.45)	−0.07 (0.09)	−0.39 (0.25)	−0.33 (0.21)
Gender						
	Male (reference)		0.00 (.)	0.00 (.)	0.00 (.)	0.00 (.)
	Female		0.15 (0.14)	−0.18 (0.10)	0.15 (0.08)	0.33 ** (0.08)
Age group						
	18–24 (reference)		0.00 (.)	0.00 (.)	0.00 (.)	0.00 (.)
	25–30		0.08 (0.38)	−0.12 (0.61)	0.14 (0.47)	0.26 (0.19)
	31–40		−0.17 (0.23)	0.37 (0.55)	0.04 (0.23)	−0.33 (0.41)
	41–50		0.09 (0.17)	0.84 (0.52)	0.28 (0.26)	−0.56 (0.39)
	51–65		−0.38 *** (0.08)	1.19 * (0.51)	0.03 (0.08)	−1.16 * (0.49)
	66–75		−0.87 * (0.35)	1.31 * (0.58)	−0.27 (0.43)	−1.57 *** (0.28)
Education, finalized						
	No finalized education (reference)		0.00 (.)	0.00 (.)	0.00 (.)	0.00 (.)
	Primary		−0.04 (0.39)	−0.40 (0.51)	−0.28 (0.42)	0.12 (0.31)
	Secondary		0.23 (0.32)	−0.26 (0.45)	0.04 (0.32)	0.29 (0.25)
	University		0.19 (0.50)	−0.25 (0.54)	−0.13 (0.38)	0.12 (0.44)
	Vocational/Technical Training		0.17 (0.53)	−0.20 (0.54)	−0.15 (0.56)	0.05 (0.54)
Household size			0.03 (0.06)	0.01 (0.04)	0.04 (0.07)	0.02 (0.03)
Average monthly sales values, last 12 months						
	KES <15,000 (reference)	<350 PPP (reference)	0.00 (.)	0.00 (.)	0.00 (.)	0.00 (.)
	KES 15,000–<45,000	350–<1040 PPP	0.48 (0.38)	−0.22 (0.27)	0.17 (0.33)	0.39 (0.41)
	KES 45,000–<75,000	1040–<1730 PPP	0.46 (0.56)	0.06 (0.16)	0.02 (0.56)	−0.03 (0.56)
	KES 75,000–<105,000	1730–<2420 PPP	0.58 (0.49)	−0.17 (0.41)	0.24 (0.56)	0.41 (0.42)
	KES 105,000–<135,000	2420–<3120 PPP	−0.17 (0.42)	0.07 (0.41)	−0.41 (0.46)	−0.48 (0.57)
	KES 135,000–<165,000	3120–<3810 PPP	0.73 (0.40)	0.27 (0.21)	0.25 (0.37)	−0.02 (0.44)
	KES >165,000	≥3810 PPP	0.01 (0.77)	−0.71 (0.57)	−0.69 (0.89)	0.02 (0.42)
Credit/loan received						
	No (reference)		0.00 (.)	0.00 (.)	0.00 (.)	0.00 (.)
	Yes		0.12 (0.20)	−0.13 (0.18)	−0.03 (0.17)	0.10 (0.20)
Constant			7.31 *** (0.71)	12.13 *** (0.90)	5.05 *** (0.64)	1.92 *** (0.38)
R-squared			0.08	0.07	0.05	0.11
N			902	902	902	902

Coefficient estimates of ordinary least square (OLS) models are shown with standard errors in parentheses. Standard errors are cluster-corrected at county level. DDS, Dietary Diversity Score; GDR, Global Dietary Recommendation; NCD, Non-communicable disease; KES, Kenyan schilling; N, number of observations. PPP conversion factor for 2023 is KES 43.29 per international dollar [44]. PPP values have been rounded to the nearest 10 for clarity. * Significant at 10% level; ** Significant at 5% level; *** Significant at 1% level.

**Table 4:** Regression results for the associations of market-related factors of traditional food markets and purchasing behavior on consumers’ DDS, GDR score, NCD-Protect, and NCD-Risk score.

Variable			DDS	GDR	NCD-Protect	NCD-Risk
Food groups bought in the market, last 30 days						
	Cereals		0.26 (0.17)	−0.02 (0.17)	0.23 (0.16)	0.25 ** (0.07)
	Roots and tubers		0.17 ** (0.06)	−0.37 (0.23)	−0.01 (0.07)	0.36 (0.18)
	Legumes, seeds and nuts		−0.20 (0.13)	0.35 * (0.16)	0.04 (0.14)	−0.31 (0.20)
	Vegetables		0.82 ** (0.20)	0.29 (0.31)	0.78 ** (0.21)	0.49 (0.33)
	Fruits		−0.24 ** (0.07)	−0.18 (0.26)	−0.35 (0.18)	−0.17 (0.22)
	Animal-source food		0.10 (0.31)	−0.20 (0.37)	0.10 (0.27)	0.30 (0.53)
	Miscellaneous		−0.15 (0.40)	−0.21 (0.28)	0.17 (0.30)	0.38 (0.36)
Travel time to the market						
	less than 5 min (reference)		0.00 (.)	0.00 (.)	0.00 (.)	0.00 (.)
	5–<10 min		−0.60 (0.36)	−0.22 (0.54)	−0.83 * (0.30)	−0.61 * (0.26)
	10–<20 min		−0.63 (0.37)	−0.48 (0.58)	−0.82 (0.38)	−0.34 (0.27)
	20–<30 min		−0.78 (0.55)	−0.55 (0.59)	−0.99 (0.56)	−0.44 ** (0.10)
	30–<60 min		−0.70 (0.43)	−0.34 (0.48)	−0.97 * (0.44)	−0.63 ** (0.22)
	60 min/1 h and more		−1.03 ** (0.27)	0.39 (0.55)	−1.08 ** (0.26)	−1.47 ** (0.47)
Frequency of market visits						
	Several times per day (reference)		0.00 (.)	0.00 (.)	0.00 (.)	0.00 (.)
	Daily, once per day		0.76 (0.47)	0.54 (0.66)	0.51 (0.52)	−0.02 (0.50)
	Every 2–3 days		0.34 (0.33)	0.47 (0.61)	0.19 (0.41)	−0.28 (0.46)
	Once per week		0.81 ** (0.19)	0.35 (0.80)	0.49 (0.38)	0.14 (0.56)
	Every two weeks		0.37 (0.23)	0.62 (0.71)	0.33 (0.42)	−0.29 (0.46)
	Once per month		0.60 (0.29)	0.27 (0.77)	0.36 (0.50)	0.09 (0.49)
	Less than once per month		0.52 (0.74)	0.05 (0.55)	0.52 (0.75)	0.47 (0.71)
Diarrhea/sickness from food, last 2 y			0.49 * (0.21)	0.51 (0.27)	0.49 (0.28)	−0.02 (0.16)
Gender						
	Male (reference)		0.00 (.)	0.00 (.)	0.00 (.)	0.00 (.)
	Female		−0.21 (0.18)	−0.45 ** (0.12)	−0.23 (0.14)	0.22 (0.19)
Age group						
	18–24 (reference)		0.00 (.)	0.00 (.)	0.00 (.)	0.00 (.)
	25–30		0.59 * (0.24)	0.74 (0.41)	0.61 (0.42)	−0.13 (0.07)
	31–40		0.65 * (0.28)	1.25 ** (0.31)	0.68 (0.45)	−0.57 ** (0.20)
	41–50		0.37 (0.26)	1.59 *** (0.30)	0.36 (0.38)	−1.24 *** (0.22)
	51–65		0.49 (0.29)	2.65 *** (0.56)	0.77 (0.49)	−1.88 *** (0.26)
	66–75		1.56 *** (0.27)	3.79 *** (0.14)	0.69 * (0.25)	−3.11 *** (0.31)
Education, finalized						
	No finalized education (reference)		0.00 (.)	0.00 (.)	0.00 (.)	0.00 (.)
	Primary		0.07 (0.24)	0.96 ** (0.31)	0.45 * (0.17)	−0.51 (0.43)
	Secondary		0.16 (0.34)	0.47 (0.30)	0.40 (0.27)	−0.07 (0.43)
	University		−0.03 (0.37)	−0.52 (0.37)	0.09 (0.30)	0.61 (0.39)
	Vocational/Technical Training		0.29 (0.24)	0.83 (0.48)	0.61 (0.30)	−0.22 (0.25)
Household size			−0.01 (0.03)	0.04 (0.06)	0.04 (0.03)	0.00 (0.05)
Money spent at the market						
	KES <100 (reference)	<3 PPP (reference)	0.00 (.)	0.00 (.)	0.00 (.)	0.00 (.)
	KES 100–<200	3–<5 PPP	−0.83 * (0.37)	−0.21 (0.66)	−0.99 * (0.38)	−0.78 (0.78)
	KES 200–<300	5–<7 PPP	−0.48 * (0.20)	0.24 (0.34)	−0.59 (0.37)	−0.83 (0.52)
	KES 300–<400	7–<9 PPP	−0.05 (0.33)	0.32 (0.49)	−0.17 (0.57)	−0.49 (0.42)
	KES 400–<500	9–<12 PPP	−0.14 (0.45)	−0.14 (0.49)	−0.52 (0.58)	−0.39 (0.74)
	KES 500–<1000	12–<23 PPP	−0.10 (0.43)	0.10 (0.47)	−0.32 (0.59)	−0.42 (0.59)
	KES ≥1000	≥23 PPP	0.10 (0.56)	−0.02 (0.26)	−0.12 (0.69)	−0.10 (0.69)
Constant			6.36 *** (0.58)	9.94 *** (0.78)	4.59 *** (0.68)	3.65 ** (1.06)
R-squared			0.11	0.17	0.10	0.15
N			835	835	835	835

Coefficient estimates of ordinary least square (OLS) models are shown with standard errors in parentheses. Standard errors are cluster-corrected at county level. DDS, Dietary Diversity Score; GDR, Global Dietary Recommendation; NCD, Non-communicable disease; KES, Kenyan schilling; N, number of observations. PPP conversion factor for 2023 is KES 43.29 per international dollar [44]. PPP values have been rounded to the nearest whole number for clarity. * Significant at 10% level; ** Significant at 5% level; *** Significant at 1% level.

## Data Availability

The data are securely stored on the servers of the Global Alliance for Improved Nutrition (GAIN). They are available upon request to the corresponding author of this paper.

## Supplementary Materials

The following supporting information can be downloaded at: https://www.mdpi.com/article/10.3390/nu17010116/s1, Table S1: Sample size calculations including 10% oversampling, by vendor and consumer for each market [69]; Table S2: Overview of diet quality indicators [42]; Table S3: Foods sold and purchased by vendors and consumers over the last 30 days, categorized into food groups; Table S4: Ordinary Logit regression results: Associations of market-related factors in traditional markets with vendors’ DDS, GDR, NCD-Protect, and NCD-Risk scores [44]; Table S5: Ordinary Logit regression results: Associations of market-related factors in traditional markets with consumers’ DDS, GDR, NCD-Protect, and NCD-Risk scores [44].

## References

[1] Ruggeri Laderchi C., Lotze-Campen H., DeClerck F., Bodirsky B., Collignon Q., Crawford M., Dietz S., Fesenfeld L., Hunecke C., Leip D. (2024). The Economics of the Food System Transformation.

[2] Development Initiatives (2022). 2022 Global Nutrition Report: Stronger Commitments for Greater Action.

[3] Kiosia A., Dagbasi A., Berkley J.A., Wilding J.P.H., Prendergast A.J., Li J.V., Swann J., Mathers J.C., Kerac M., Morrison D. (2024). The Double Burden of Malnutrition in Individuals: Identifying Key Challenges and Re-Thinking Research Focus. Nutr. Bull..

[4] Downs S.M., Ahmed S., Fanzo J., Herforth A. (2020). Food Environment Typology: Advancing an Expanded Definition, Framework, and Methodological Approach for Improved Characterization of Wild, Cultivated, and Built Food Environments toward Sustainable Diets. Foods.

[5] HLPE (2017). Nutrition and Food Systems.

[6] Turner C., Aggarwal A., Walls H., Herforth A., Drewnowski A., Coates J., Kalamatianou S., Kadiyala S. (2018). Concepts and Critical Perspectives for Food Environment Research: A Global Framework with Implications for Action in Low- and Middle-Income Countries. Glob. Food Secur..

[7] Cook B., Trevenen-Jones A., Sivasubramanian B. (2024). Nutritional, Economic, Social and Governance Implications of Traditional Food Markets for Vulnerable Populations in Sub-Saharan Africa: A Systematic Narrative Review. Front. Sustain. Food Syst..

[8] Hannah C., Davies J., Green R., Zimmer A., Anderson P., Battersby J., Baylis K., Joshi N., Evans T.P. (2022). Persistence of Open-Air Markets in the Food Systems of Africa’s Secondary Cities. Cities.

[9] Odeyale T.O. (2020). Actor-Network, Conflict and the Commodification of Planning: Role of Traditional Food Markets in Shaping the Built Environment. Habitat Int..

[10] Gupta N., Deshmukh V., Verma S., Puri S., Tandon N., Arora N.K. (2023). Food Environment Framework in Low- and Middle-Income Countries—An Integrative Review. Glob. Food Secur..

[11] Popkin B.M. (2014). Nutrition, Agriculture and the Global Food System in Low and Middle Income Countries. Food Policy.

[12] Demmler K.M., Ecker O., Qaim M. (2018). Supermarket Shopping and Nutritional Outcomes: A Panel Data Analysis for Urban Kenya. World Dev..

[13] de Kanter E., Trevenen-Jones A., Billiard C.M.J. (2024). Nutrition Security and Traditional Food Markets in Africa: Gender Insights. Front. Sustain..

[14] Demmler K. (2020). The Role of Small and Medium-Sized Enterprises in Nutritious Food Supply Chains in Africa.

[15] Downs S.M., Fox E.L., Mutuku V., Muindi Z., Fatima T., Pavlovic I., Husain S., Sabbahi M., Kimenju S., Ahmed S. (2022). Food Environments and Their Influence on Food Choices: A Case Study in Informal Settlements in Nairobi, Kenya. Nutrients.

[16] Simon S. (2007). Promises and Challenges of the Informal Food Sector in Developing Countries.

[17] Nordhagen S., Lee J., Onuigbo-Chatta N., Okoruwa A., Monterrosa E., Lambertini E., Pelto G.H. (2022). What Is Safe and How Much Does It Matter? Food Vendors’ and Consumers’ Views on Food Safety in Urban Nigeria. Foods.

[18] Cortese R.D.M., Veiros M.B., Feldman C., Cavalli S.B. (2016). Food Safety and Hygiene Practices of Vendors during the Chain of Street Food Production in Florianopolis, Brazil: A Cross-Sectional Study. Food Control.

[19] Resnick D. (2017). Chapter 6. Governance: Informal Food Markets in Africa’s Cities. IFPRI Book Chapters.

[20] Herforth A., Ahmed S. (2015). The Food Environment, Its Effects on Dietary Consumption, and Potential for Measurement within Agriculture-Nutrition Interventions. Food Secur..

[21] Turner C., Kalamatianou S., Drewnowski A., Kulkarni B., Kinra S., Kadiyala S. (2020). Food Environment Research in Low- and Middle-Income Countries: A Systematic Scoping Review. Adv. Nutr..

[22] Pradeilles R., Irache A., Wanjohi M.N., Holdsworth M., Laar A., Zotor F., Tandoh A., Klomegah S., Graham F., Muthuri S.K. (2021). Urban Physical Food Environments Drive Dietary Behaviours in Ghana and Kenya: A Photovoice Study. Health Place.

[23] Wanjohi M.N., Pradeilles R., Asiki G., Holdsworth M., Kimani-Murage E.W., Muthuri S.K., Irache A., Laar A., Zotor F., Tandoh A. (2023). Community Perceptions on the Factors in the Social Food Environment That Influence Dietary Behaviour in Cities of Kenya and Ghana: A Photovoice Study. Public Health Nutr..

[24] Ambikapathi R., Shively G., Leyna G., Mosha D., Mangara A., Patil C.L., Boncyk M., Froese S.L., Verissimo C.K., Kazonda P. (2021). Informal Food Environment Is Associated with Household Vegetable Purchase Patterns and Dietary Intake in the DECIDE Study: Empirical Evidence from Food Vendor Mapping in Peri-Urban Dar Es Salaam, Tanzania. Glob. Food Secur..

[25] Downs S.M., Fox E.L., Zivkovic A., Mavros T., Sabbahi M., Merchant E.V., Mutuku V., Okumu-Camerra K., Kimenju S. (2022). Drivers of Food Choice among Women Living in Informal Settlements in Nairobi, Kenya. Appetite.

[26] Westbury S., Ghosh I., Jones H.M., Mensah D., Samuel F., Irache A., Azhar N., Al-Khudairy L., Iqbal R., Oyebode O. (2021). The Influence of the Urban Food Environment on Diet, Nutrition and Health Outcomes in Low-Income and Middle-Income Countries: A Systematic Review. BMJ Glob. Health.

[27] Marshall Q., Fanzo J., Barrett C.B., Jones A.D., Herforth A., McLaren R. (2021). Building a Global Food Systems Typology: A New Tool for Reducing Complexity in Food Systems Analysis. Front. Sustain. Food Syst..

[28] The World Bank Middle Income Countries. https://www.worldbank.org/en/country/mic/overview.

[29] Global Alliance for Improved Nutrition (GAIN), Johns Hopkins University (2020). The Food Systems Dashboard.

[30] KNBS (2016). Kenya Integrated Household Budget Survey 2015–2016.

[31] Kenya National Bureau of Statistics (2019). 2019 Kenya Population and Housing Census: Volume II.

[32] Kenya National Bureau of Statistics (2024). Poverty Report: Based on the 2022 Kenya Continuous Household Survey.

[33] Global Diet Quality Project DQQ for Kenya. https://drive.google.com/drive/folders/12CBmuQai7B70r7Xp8TBSeAoWL1HWY_og.

[34] Demmler K., Friesen V., Neufeld L., Caballero B. (2022). Food Security and Nutrition Surveillance in Low- and Middle-Income Countries. Encyclopedia of Human Nutrition (Third Edition, Updated).

[35] Uyar B.T.M., Talsma E.F., Herforth A.W., Trijsburg L.E., Vogliano C., Pastori G., Bekele T.H., Huong L.T., Brouwer I.D. (2023). The DQQ Is a Valid Tool to Collect Population-Level Food Group Consumption Data: A Study Among Women in Ethiopia, Vietnam, and Solomon Islands. J. Nutr..

[36] Herforth A.W., Ballard T., Rzepa A. (2024). Development of the Diet Quality Questionnaire for Measurement of Dietary Diversity and Other Diet Quality Indicators. Curr. Dev. Nutr..

[37] Women’s Dietary Diversity Project (WDDP) Study Group (2017). Development of a Dichotomous Indicator for Population-Level Assessment of Dietary Diversity in Women of Reproductive Age. Curr. Dev. Nutr..

[38] Kant A.K., Block G., Schatzkin A., Ziegler R.G., Nestle M. (1991). Dietary Diversity in the US Population, NHANES II, 1976–1980. J. Am. Diet. Assoc..

[39] Torheim L.E., Ouattara F., Diarra M.M., Thiam F.D., Barikmo I., Hatløy A., Oshaug A. (2004). Nutrient Adequacy and Dietary Diversity in Rural Mali: Association and Determinants. Eur. J. Clin. Nutr..

[40] Vandevijvere S., De Vriese S., Huybrechts I., Moreau M., Van Oyen H. (2010). Overall and Within-Food Group Diversity Are Associated with Dietary Quality in Belgium. Public Health Nutr..

[41] World Health Organization (2018). Healthy Diet. Fact Sheet No. 394.

[42] Global Diet Quality Project Diet Quality Questionnaire (DQQ) (2023). Indicator Guide Version 11. https://drive.google.com/file/d/1eplRm9i5_109-a5Ac1Lqj-lUI3VgVIFx/view.

[43] Wooldridge J.M. (2016). Introductory Econometrics: A Modern Approach.

[44] The World Bank Group PPP Conversion Factor, GDP (LCU per International $)—Kenya. https://data.worldbank.org.

[45] Kimenju S.C., Barling D., Fanzo J. (2018). Chapter Three-Ultra-Processed Foods and Obesity in Central Kenya. Advances in Food Security and Sustainability.

[46] Demmler K.M., Klasen S., Nzuma J.M., Qaim M. (2017). Supermarket Purchase Contributes to Nutrition-Related Non-Communicable Diseases in Urban Kenya. PLoS ONE.

[47] Phelps N.H., Singleton R.K., Zhou B., Heap R.A., Mishra A., Bennett J.E., Paciorek C.J., Lhoste V.P., Carrillo-Larco R.M., Stevens G.A. (2024). Worldwide Trends in Underweight and Obesity from 1990 to 2022: A Pooled Analysis of 3663 Population-Representative Studies with 222 Million Children, Adolescents, and Adults. Lancet.

[48] (2021). Global Diet Quality Project Kenya. DQQ Data. https://dataverse.harvard.edu/dataverse/dqqdata.

[49] Namugumya B.S., Muyanja C. (2012). Contribution of Street Foods to the Dietary Needs of Street Food Vendors in Kampala, Jinja and Masaka Districts, Uganda. Public Health Nutr..

[50] Gupta S., Sunder N., Pingali P.L. (2020). Market Access, Production Diversity, and Diet Diversity: Evidence From India. Food Nutr. Bull..

[51] Sibhatu K.T., Krishna V.V., Qaim M. (2015). Production Diversity and Dietary Diversity in Smallholder Farm Households. Proc. Natl. Acad. Sci. USA.

[52] Nandi R., Nedumaran S., Ravula P. (2021). The Interplay between Food Market Access and Farm Household Dietary Diversity in Low and Middle Income Countries: A Systematic Review of Literature. Glob. Food Secur..

[53] Sekabira H., Nansubuga Z., Ddungu S.P., Nazziwa L. (2022). Farm Production Diversity, Household Dietary Diversity, and Nutrition: Evidence from Uganda’s National Panel Survey. PLoS ONE.

[54] Appelhans B.M., French S.A., Tangney C.C., Powell L.M., Wang Y. (2017). To What Extent Do Food Purchases Reflect Shoppers’ Diet Quality and Nutrient Intake?. Int. J. Behav. Nutr. Phys. Act..

[55] Duran A.C., de Almeida S.L., Latorre M.d.R.D., Jaime P.C. (2016). The Role of the Local Retail Food Environment in Fruit, Vegetable and Sugar-Sweetened Beverage Consumption in Brazil. Public Health Nutr..

[56] Vedovato G.M., Trude A.C.B., Kharmats A.Y., Martins P.A. (2015). Degree of Food Processing of Household Acquisition Patterns in a Brazilian Urban Area Is Related to Food Buying Preferences and Perceived Food Environment. Appetite.

[57] Heiman M.L., Greenway F.L. (2016). A Healthy Gastrointestinal Microbiome Is Dependent on Dietary Diversity. Mol. Metab..

[58] Development Initiatives Kenya Country Nutrition Profile. https://globalnutritionreport.org/nutrition-profiles/africa/eastern-africa/kenya/.

[59] Visser J., Wangu J. (2021). Women’s Dual Centrality in Food Security Solutions: The Need for a Stronger Gender Lens in Food Systems’ Transformation. Curr. Res. Environ. Sustain..

[60] de Andrade S.C., Previdelli Á.N., Cesar C.L.G., Marchioni D.M.L., Fisberg R.M. (2016). Trends in Diet Quality among Adolescents, Adults and Older Adults: A Population-Based Study. Prev. Med. Rep..

[61] Global Alliance for Improved Nutrition (2022). Review of Food Safety Training in Low- and Middle-Income Countries.

[62] Grace D., Dipeolu M., Alonso S. (2019). Improving Food Safety in the Informal Sector: Nine Years Later. Infect. Ecol. Epidemiol..

[63] HLPE (2024). Strengthening Urban and Peri-Urban Food Systems to Achieve Food Security and Nutrition, in the Context of Urbanization and Rural Transformation.

[64] Hülsen V., Khonje M.G., Qaim M. (2024). Market Food Environments and Child Nutrition. Food Policy.

[65] Halliday J., Platenkamp L., Nicolarea Y. (2019). Menu of Actions to Shape Urban Food Environments for Improved Nutrition.

[66] Takeshima H., Yamauchi F., Edeh H.O., Hernandez M.A. (2023). Solar-Powered Cold-Storage and Agrifood Market Modernization in Nigeria. Agric. Econ..

[67] Crosbie E., Alvarez M.G.O., Cao M., Renteria L.S.V., Rodriguez E., Flota A.L., Carriedo A. (2023). Implementing Front-of-Pack Nutrition Warning Labels in Mexico: Important Lessons for Low- and Middle-Income Countries. Public Health Nutr..

[68] Drewnowski A., Monterrosa E.C., de Pee S., Frongillo E.A., Vandevijvere S. (2020). Shaping Physical, Economic, and Policy Components of the Food Environment to Create Sustainable Healthy Diets. Food Nutr. Bull..

[69] Uakarn C., Chaokromthong K., Sintao N. (2021). Sample Size Estimation Using Yamane and Cochran and Krejcie and Morgan and Green Formulas and Cohen Statistical Power Analysis by G*Power and Comparisons. APHEIT Int. J..

