# Supplementary material for: Food Environments and Diet Quality Among Vendors and Consumers in Five Traditional Urban Markets in Kenya
Source: Nutrients. 2024 Dec 30;17(1):116. doi: 10.3390/nu17010116 (PMC11723231; doi:10.3390/nu17010116)
Supplement: Supplementary file 1 [file nutrients-17-00116-s001.zip › nutrients-3369605-supplementary.pdf]

Supplementary files

Random sampling techniques were applied with the aim to select representative samples of vendors and consumers from all five market areas. The statistical unit for this study was the individual vendor/consumer in the respective market. To determine the sample sizes for both vendor and consumer, the below formular [69] and assumptions have been used:

1. n = z2 * p(1-p)/e2
2. n′ = n/(1 + ((z2 * p(1-p))/(e2 N)))

N: is the estimated average population size of vendors and consumers on market and non-market days by the market committees in the respective markets.

z: Z score = 1.44 for a confidence level of 85% (used for vendor) / 1.28 for a confidence level of 80% (used for consumer)

e: is the margin of error of 5%

p: is the population proportion 50%

Table S1 specifies the sample sizes for vendors and consumers for which the probability that the true value falls within a specific range of values (CI= 85%/80%). The provided target numbers include a 8-10% oversample, accounting for possible attrition.

Table S1. Sample size calculations including 10% oversampling, by vendor and consumer for each market.

	**Vendor (CI 85%)**	**Consumer (CI 80%)**
Marikiti Market (Machakos)*	215	140
Free Area Market (Nakuru)	157	153
Thika Town Madaraka Market (Kiambu)	203	169
Kongowea Market (Mombasa)	218	169
Soweto Market (Nairobi)	171	175
**Total**	**964**	**807**

CI: Confidence Interval. *Sample size calculation for Machakos included an oversample of 8% and sample size calculation for consumers was based on CI 75%.

Reference

[69] Uakarn, C.; Chaokromthong, K.; Sintao, N. Sample Size Estimation Using Yamane and Cochran and Krejcie and Morgan and Green Formulas and Cohen Statistical Power Analysis by G*Power and Comparisons. APHEIT Int. J. 2021, 10, 76–86.

Table S2. Overview of diet quality indicators.

**Diet quality indicators, based on the DQQ [42]**	**Range**	**Description**
Dietary Diversity Score (DDS)	0 - 10	Positive, semi-continuous score of food group diversity, expressed as the average score out of 10.
Global Dietary Recommendations (GDR) score	0 - 18	Positive, semi-continuous score that indicates compliance with global dietary recommendations, calculated: NCD-Protect – NCD-Risk + 9.
NCD-Protect score	0 - 9	Positive, semi-continuous score of dietary factors protective against NCDs, sub-component of GDR score.
NCD-Risk score	0 - 9	Negative, semi-continuous score (0-9) of dietary factors for NCDs, proxy for ultra-processed food intake, sub-component of GDR score

DQQ: Diet Quality Questionnaire; NCD: non-communicable disease.

Reference

[42] Global Diet Quality Project Diet Quality Questionnaire (DQQ). Indicator Guide Version 11 2023. https://drive.google.com/file/d/1eplRm9i5_109-a5Ac1Lqj-lUI3VgVIFx/view

Table S3. Foods sold and purchased by vendors and consumers over the last 30 days, categorized into seven food groups.

Food groups	Foods included
Cereals	Rice, wheat, maize flour, bread, pasta
Roots and tubers	Roots and tubers
Legumes, seeds and nuts	Legumes, seeds and nuts
Vegetables	Dark green leafy vegetables, vitamin A rich orange vegetables, and other vegetables
Fruits	Vitamin A rich orange fruits, and other fruits
Animal source food	Milk and dairy, eggs, poultry, beef, goat, lamb, pig, fish and seafood
Miscellaneous	Culinary ingredients (oil, fats, lard, butter, sugar, salt, spices, tea leaves), bottled water, beverages (juices, soft drinks, alcoholic drinks), sweets (cake, cookies, biscuits, chocolate), deep fried and/or salty snacks (chips, mandazis, samosas)

Table S4. Ordinary Logit regression results: Associations of market-related factors in traditional markets with vendors' DDS, GDR score, NCD-Protect and NCD-Risk score.

	**DDS**	**GDR**	**NCD-Protect**	**NCD-Risk**
**Variable**	b/se	b/se	b/se	b/se
**Food groups sold in the market, last 30 days**				
Cereals	-0.46 (0.49)	0.16 (0.34)	-0.09 (0.47)	-0.36* (0.21)
Roots and tubers	0.22* (0.12)	0.32 (0.22)	0.29** (0.15)	-0.08 (0.30)
Legumes, seeds & nuts	-0.02 (0.20)	0.08 (0.26)	0.08 (0.23)	0.09 (0.18)
Vegetables	0.05 (0.16)	0.22 (0.23)	0.17 (0.15)	-0.04 (0.21)
Fruits	-0.09 (0.12)	0.08 (0.21)	0.14 (0.14)	0.13 (0.10)
Animal source food	0.30 (0.20)	0.59*** (0.15)	0.50** (0.24)	-0.37 (0.40)
Miscellaneous (ingredients, sweets, snacks)	-0.05 (0.67)	0.51 (0.85)	0.20 (0.54)	-0.30 (0.33)
**Travel time to the market**				
less than 5 mins (*reference*)	0.00 (.)	0.00 (.)	0.00 (.)	0.00 (.)
5 - <10 min	-1.04*** (0.33)	-0.12 (0.48)	-0.44 (0.35)	-0.36 (0.22)
10 - <20 min	-1.00*** (0.31)	-0.39 (0.46)	-0.50 (0.33)	-0.23 (0.18)
20 - <30 min	-0.45 (0.28)	-0.06 (0.37)	0.15 (0.30)	-0.09 (0.27)
30 - <60 mins	-1.05** (0.41)	-0.37 (0.42)	-0.73* (0.41)	-0.46 (0.28)
60 min/1hr and more	-0.97** (0.38)	-0.30 (0.49)	-0.47 (0.38)	-0.15 (0.25)
**Diarrhea/sickness (from food), last 30 days**	-0.48 (0.41)	-0.06 (0.08)	-0.34 (0.22)	-0.18 (0.29)
**Gender**				
Male (*reference*)	0.00 (.)	0.00 (.)	0.00 (.)	0.00 (.)
Female	0.11 (0.13)	-0.15* (0.09)	0.10 (0.09)	0.28*** (0.10)
**Age group**				
18-24 (*reference*)	0.00 (.)	0.00 (.)	0.00 (.)	0.00 (.)
25-30	0.18 (0.43)	-0.07 (0.54)	0.14 (0.49)	0.21 (0.17)
31-40	-0.01 (0.24)	0.29 (0.50)	0.12 (0.24)	-0.40 (0.43)
41-50	0.24 (0.20)	0.70 (0.45)	0.29 (0.26)	-0.73* (0.38)
51-65	-0.30*** (0.10)	1.05** (0.47)	0.08 (0.09)	-1.34*** (0.51)
66-75	-0.93** (0.39)	0.95* (0.51)	-0.43 (0.44)	-1.82*** (0.38)
**Education, finalized**				
No finalized education (*reference*)	0.00 (.)	0.00 (.)	0.00 (.)	0.00 (.)

Primary		0.00 (0.37)	-0.34 (0.45)	-0.22 (0.37)	0.03 (0.25)
Secondary		0.29 (0.26)	-0.23 (0.38)	0.06 (0.27)	0.20 (0.13)
University		0.30 (0.45)	-0.40 (0.47)	-0.14 (0.32)	0.04 (0.37)
Vocational/Technical Training		0.14 (0.48)	-0.11 (0.41)	-0.20 (0.50)	-0.10 (0.48)
**Household size**		0.02 (0.05)	0.01 (0.04)	0.02 (0.06)	0.04 (0.03)
**Average monthly sales values, last 12 months** [a]					
< 15,000 KES (*reference*)	<350 PPP (*reference*)	0.00 (.)	0.00 (.)	0.00 (.)	0.00 (.)
15,000 - < 45,000 KES	350 - <1,040 PPP	0.36 (0.40)	-0.13 (0.27)	0.07 (0.36)	0.53 (0.42)
45,000 - < 75,000 KES	1,040 - <1,730 PPP	0.36 (0.59)	0.15 (0.16)	-0.01 (0.61)	0.06 (0.65)
75,000 - < 105,000 KES	1,730 - <2,420 PPP	0.41 (0.54)	-0.14 (0.40)	0.07 (0.57)	0.59 (0.54)
105,000 - < 135,000 KES	2,420 - <3,120 PPP	-0.40 (0.47)	0.11 (0.37)	-0.52 (0.51)	-0.36 (0.69)
135,000 - < 165,000 KES	>3,120 PPP [b]	0.54 (0.39)	0.25 (0.20)	0.16 (0.42)	0.36 (0.54)
> 165,000 KES		-0.22 (0.86)	-0.52 (0.59)	-0.81 (0.91)	0.29 (0.46)
**Credit/loan received**					
No (*reference*)		0.00 (.)	0.00 (.)	0.00 (.)	0.00 (.)
Yes		0.15 (0.22)	-0.09 (0.18)	-0.02 (0.16)	0.15 (0.22)
**Pseudo R-squared**		0.02	0.02	0.01	0.03
**N**		902	902	902	902

Estimates are shown based on an ordinary logit regression with standard errors in parentheses. Standard errors are cluster-corrected at county level. DDS, Dietary Diversity Score; GDR, Global Dietary Recommendation; KES, Kenyan schilling; N, number of observations. [a] PPP conversion factor for 2023 is 43.29 KES per international dollar [44]. PPP values have been rounded to the nearest 10 for clarity. * Significant at 10% level; ** Significant at 5% level; *** Significant at 1% level.

Reference

[44] The World Bank Group PPP Conversion Factor, GDP (LCU per International $) - Kenya Available online: https://data.worldbank.org (accessed on 18 December 2024).

Table S5. Ordinary Logit regression results: Associations of market-related factors in traditional markets with consumers' DDS, GDR score, NCD-Protect and NCD-Risk score.

Variable	DDS	GDR	NCD-Protect	NCD-Risk
**Food groups bought in the market, last 30 days**	b/se	b/se	b/se	b/se
Cereals	0.41** (0.21)	-0.05 (0.11)	0.28* (0.16)	0.19** (0.08)
Roots and tubers	0.22*** (0.08)	-0.30 (0.19)	-0.05 (0.09)	0.28* (0.15)
Legumes, seeds & nuts	-0.28* (0.15)	0.31*** (0.11)	0.10 (0.15)	-0.23 (0.20)
Vegetables	0.83*** (0.17)	0.32 (0.29)	0.76*** (0.27)	0.48 (0.32)
Fruits	-0.33*** (0.10)	-0.16 (0.22)	-0.41* (0.23)	-0.09 (0.14)
Animal source food	0.19 (0.34)	-0.13 (0.30)	0.15 (0.25)	0.26 (0.42)
Miscellaneous (ingredients, sweets, snacks)	0.10 (0.37)	-0.17 (0.20)	0.29 (0.29)	0.22 (0.34)
**Travel time to the market**				
less than 5 mins (*reference*)	0.00 (.)	0.00 (.)	0.00 (.)	0.00 (.)
5 - <10 min	-0.69** (0.34)	-0.10 (0.54)	-0.98*** (0.20)	-0.54** (0.21)
10 - <20 min	-0.71** (0.34)	-0.32 (0.62)	-0.95*** (0.32)	-0.33 (0.22)
20 - <30 min	-0.81 (0.50)	-0.39 (0.61)	-1.03** (0.46)	-0.32*** (0.11)
30 - <60 mins	-0.69* (0.40)	-0.13 (0.54)	-1.03*** (0.37)	-0.59*** (0.19)
60 min/1hr and more	-1.12** (0.50)	0.45 (0.55)	-1.02** (0.43)	-1.41** (0.56)
**Frequency of market visits**				
Several times per day (*reference*)	0.00 (.)	0.00 (.)	0.00 (.)	0.00 (.)
Daily, once per day	0.94 (0.62)	0.31 (0.59)	0.64 (0.67)	0.13 (0.50)
Every 2-3 days	0.52 (0.43)	0.24 (0.56)	0.25 (0.53)	-0.12 (0.40)
Once per week	1.07*** (0.31)	0.18 (0.70)	0.63 (0.52)	0.26 (0.50)
Every two weeks	0.60** (0.30)	0.40 (0.67)	0.45 (0.54)	-0.07 (0.45)
Once per month	0.92** (0.46)	0.13 (0.72)	0.57 (0.69)	0.31 (0.44)
Less than once per month	0.73 (0.97)	-0.04 (0.58)	0.73 (0.93)	0.49 (0.64)
**Diarrhea/sickness (from food), last 2 years**	0.59*** (0.20)	0.33 (0.25)	0.59* (0.33)	0.09 (0.11)

**Gender**					
Male (*reference*)		0.00 (.)	0.00 (.)	0.00 (.)	0.00 (.)
Female		-0.27 (0.24)	-0.37*** (0.08)	-0.26 (0.18)	0.13 (0.17)
**Age group**					
18-24 (*reference*)		0.00 (.)	0.00 (.)	0.00 (.)	0.00 (.)
25-30		0.60*** (0.20)	0.60** (0.27)	0.56 (0.39)	-0.10 (0.08)
31-40		0.70*** (0.23)	1.02*** (0.19)	0.65 (0.42)	-0.50*** (0.19)
41-50		0.35 (0.23)	1.35*** (0.18)	0.27 (0.33)	-1.08*** (0.24)
51-65		0.48** (0.24)	2.32*** (0.45)	0.73 (0.45)	-1.67*** (0.29)
66-75		2.29*** (0.28)	3.40*** (0.40)	0.63*** (0.18)	-3.29*** (0.40)
**Education, finalized**					
No finalized education (*reference*)		0.00 (.)	0.00 (.)	0.00 (.)	0.00 (.)
Primary		0.08 (0.28)	0.82*** (0.26)	0.54*** (0.15)	-0.43 (0.35)
Secondary		0.19 (0.40)	0.42 (0.27)	0.46 (0.28)	-0.14 (0.36)
University		-0.11 (0.44)	-0.40 (0.34)	0.11 (0.31)	0.44 (0.34)
Vocational/Technical Training		0.24 (0.28)	0.70 (0.45)	0.68** (0.33)	-0.19 (0.19)
**Household size**		-0.01 (0.04)	0.03 (0.05)	0.03 (0.03)	0.01 (0.05)
**Money spend at the market** [a]					
<100 KES (*reference*)	<3 PPP (*reference*)	0.00 (.)	0.00 (.)	0.00 (.)	0.00 (.)
100 - <200 KES	3 - <5 PPP	-1.06** (0.46)	-0.10 (0.68)	-1.03* (0.59)	-0.62 (0.60)
200 - <300 KES	5 - <7 PPP	-0.78 (0.49)	0.27 (0.33)	-0.76 (0.64)	-0.71** (0.33)
300 - <400 KES	7 - <9 PPP	-0.27 (0.57)	0.36 (0.37)	-0.31 (0.87)	-0.43 (0.30)
400 - <500 KES	9 - <12 PPP	-0.49 (0.67)	-0.05 (0.39)	-0.72 (0.87)	-0.23 (0.55)
500 - <1000 KES	12 - <23 PPP	-0.40 (0.69)	0.14 (0.35)	-0.43 (0.90)	-0.30 (0.44)
≥1000 KES	≥23 PPP	-0.11 (0.84)	-0.00 (0.21)	-0.24 (1.00)	-0.10 (0.55)
**Pseudo R-squared**		0.03	0.04	0.03	0.04
**Number of observations**		835	835	835	835

Estimates are shown based on an ordinary logit regression with standard errors in parentheses. Standard errors are cluster-corrected at county level. DDS, Dietary Diversity Score; GDR, Global Dietary Recommendation; KES, Kenyan schilling; N, number of observations. [a] PPP conversion factor for 2023 is 43.29 KES per international dollar [44]. PPP values have been rounded to the nearest whole number for clarity. * Significant at 10% level; ** Significant at 5% level; *** Significant at 1% level.

Reference

[44] The World Bank Group PPP Conversion Factor, GDP (LCU per International $) - Kenya Available online: https://data.worldbank.org (accessed on 18 December 2024).
